# Finite Element Modelling and Experimental Validation of the Enamel Demineralisation Process at the Rod Level

**DOI:** 10.1016/j.jare.2020.08.018

**Published:** 2020-09-06

**Authors:** Enrico Salvati, Cyril Besnard, Robert A. Harper, Thomas Moxham, Richard M. Shelton, Gabriel Landini, Alexander M. Korsunsky

**Affiliations:** a MBLEM, Department of Engineering Science, University of Oxford, Parks Road, Oxford OX13PJ, UK; b Polytechnic Department of Engineering and Architecture (DPIA), University of Udine, Via delle Scienze 208, Udine 33100, Italy; c School of Dentistry, College of Medical and Dental Sciences, University of Birmingham, 5 Mill Pool Way, Edgbaston, Birmingham B5 7EG, UK; d Diamond Light Source, Harwell Science and Innovation Campus, Harwell OX11 0DE, UK

**Keywords:** Dental demineralisation, Reaction-diffusion, Synchrotron CT, Demineralisation simulation, FEM, Enamel

## Abstract

In the past years, a significant amount of effort has been directed at the observation and characterisation of caries using experimental techniques. Nevertheless, relatively little progress has been made in numerical modelling of the underlying demineralisation process.

The present study is the first attempt to provide a simplified calculation framework for the numerical simulation of the demineralisation process at the length scale of enamel rods and its validation by comparing the data with statistical analysis of experimental results.

FEM model was employed to simulate a time-dependent reaction-diffusion equation process in which H ions diffuse and cause demineralisation of the enamel. The local orientation of the hydroxyapatite crystals was taken into account. Experimental analysis of the demineralising front was performed using advanced high-resolution synchrotron X-ray micro-Computed Tomography. Further experimental investigations were conducted by means of SEM and STEM imaging techniques.

Besides establishing and validating the new modelling framework, insights into the role of the etchant solution pH level were obtained. Additionally, some light was shed on the origin of different types of etching patterns by simulating the demineralisation process at different etching angles of attack.

The implications of this study pave the way for simulations of enamel demineralisation within different complex scenarios and across the range of length scales. Indeed, the framework proposed can incorporate the presence of chemical species other than H ions and their diffusion and reaction leading to dissolution and re-precipitation of hydroxyapatite. It is the authors’ hope and aspiration that ultimately this work will help identify new ways of controlling and preventing caries.

## Graphical abstract

**Figure:**
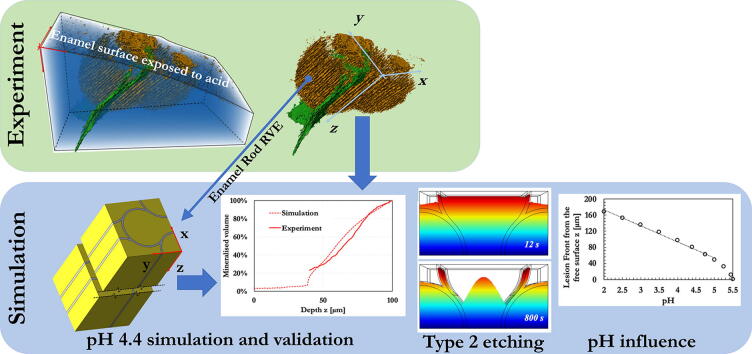

## Introduction

Dental caries is a major public health problem that affects people of all ages around the world. The continued demand for dental treatment and disease prevention has led to a great need for a deeper understanding of the underlying mechanism causing caries, namely, the mineral dissolution of the complex hierarchical structure of human teeth [1]. Typically, dental caries begins at the outermost layer of teeth (enamel), which is a highly mineralised substance that covers the crown portion of the teeth and serves as the wear resistant part of teeth. Although it is worth mentioning that caries may originate in cementum and dentine of the tooth as well [2]. At the microscopic scale, enamel consists of tightly packed keyhole-shaped *rods* approximately 5 µm in width) aligned approximately perpendicular to the dentin outwards to the surface [3].

**Fig. 1:**
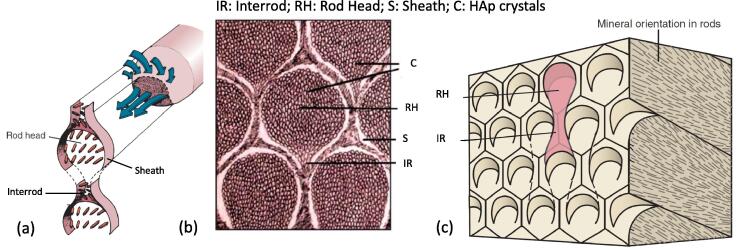
Schematisation and evidence of the enamel structure at the micro-scale – a collection of images from the literature. (a) The left side of the diagram shows orientation of crystals in the forming rod head and tail, while the right part shows how forming crystals pack in the rod from the cell complex [5]. (b) Image showing the different HAp crystals in the rod head, interrod and sheath domains [4]. (c) Diagram of the six-sided ameloblasts overlying keyhole-shaped enamel rods [9].

Within each rod, it is possible to identify a region occupying a minor area that is generally called *interrod enamel*. Therefore, the remaining region is named as *rod head.* These rods are primarily composed of Hydroxyapatite (HAp) crystals (approximately 25–30 nm thick and 200 nm long), which are covered by a nanometre-thin layer of enamelin. It is important to note that there exists no discontinuity between rod head and interrod regions as the HAp crystals gradually change their orientation from one region to the other. This interrod enamel shows a more prominent gradient in HAp crystal organisation, particularly its orientation. An interface between the rod enamel can also be identified, which is termed the *rod sheath.* The rod sheath is a highly organic domain, containing substances like enamel protein. It is thought to be acid-resistant and in some cases can be absent. Its thickness may vary from 0 to hundreds of nanometre nm, depending on the size of HAp crystal at the border between two adjacent rods [4]. An overview on the structural organisation of the enamel al this length scale is shown in Fig. 1(a-b)*.* In general, enamel shows 3%–4% content of water by weight, a high fraction of other inorganic matter (92%–96%) and its remaining composition is 1%–2% organic material.

The crystallisation of the HAp is carried out by *ameloblasts* during tooth development, well in advance of tooth eruption. These cells are generally of columnar shape, with approximately hexagonal cross section and their size directly reflects the final size of the enamel rods [5], [6], as depicted in Fig. 1. The orientation of these crystallites (or crystals) varies across the rod cross-section, but is predominantly aligned with the longitudinal direction of the rod [7], particularly within the rod head region. As concerns the rod sheath, this material contains relatively more protein than other areas of enamel. For this reason, the rod sheath is characterized as being hypo-mineralised in comparison with the rest of the highly mineralised enamel. For the sake of the present study, it is worth describing also how HAp crystals are arranged within the layer of the enamel approaching the free surface. It has been shown that an *aprismatic* layer is present on the outermost surface of the enamel characterised by a laminate-like structure. The aprismatic layer is deposited by the ameloblasts each day of life, ant it is thought to occur predominantly during the diurnal hours, and for this reason the aprismatic layer appears to be periodically repeating forming this laminate look [8]. Within the superficial aprismatic layer, HAp crystals are well-aligned parallel to each other and perpendicular to the surface [1], and this characteristic plays a role on the demineralisation process.

Overall, the enamel shows a hierarchical structure, starting at the nano-scale with HAp crystallites being the main constituents. At a higher hierarchical level (micron-scale) the length of reference is the enamel rod. While at the macroscopic level (millimetre-scale or Shmelzmuster level), bundles of enamel rods are considered to build up the actual geometrical shape of the tooth that is visible with bare eyes.

Structurally, the early enamel caries process is thought of as enamel density reduction and removal by demineralisation in the presence of acids. In natural caries, demineralisation is primarily driven by the products of the anaerobic metabolism of cariogenic bacteria within dental plaque producing acid. In general, the enamel demineralisation progression is mediated by the acid diffusion into enamel causing the dissolution of HAp crystals. The most prominent properties of the acidic solution in terms of affecting the demineralisation progress are: the acid type, the pH, the acid concentration, the acid titration, the extent of enamel surface exposure to the acid and the exposure time [1].

The rate and directionality of enamel demineralisation at the millimetre scale is thought to be mainly driven by the rod orientation with respect to the acid wet surface. To understand the influence of the preferential demineralising direction it is of paramount importance to consider the anisotropic diffusivity properties of a given etching agent within the HAp crystals [10]. These crystals show a distinct preferred diffusion direction which means that the progress of diffusion depends mainly on the crystal orientation distribution (texture) within the enamel. For this reason, the advancement of the demineralisation front appears to be heterogeneous even at higher length scales, i.e. millimetre and the micrometre scales [11].

This heterogeneous structural organisation of the HAp crystals gives rise to different patterns of etching at the rod enamel scale. As described by Silverstone et al. [12], when the enamel is exposed to lactic acid, three different etching types can be identified. i) Type 1: a preferential loss is observed in the rod enamel core; ii) Type 2: the enamel rod appears largely intact compared with the mineral loss observed at its periphery; and iii) Type 3: a random pattern is observed that cannot be correlated with the shape of the enamel rods. Whether etching of Type 1, 2 or 3 is realised for a given agent is thought to depend on the difference in the orientation of HAp crystals relative to the direction of acid attack, although no systematic evidence or interpretation has been shown so far. However, it is clear that different etchant solutions react differently on different faces of the crystals, so that the nature of acidic chemical species plays a significant role in determining the etching type.

The purpose of this work is to create a simple and reliable mathematical descriptive model for the prediction of the enamel demineralisation process at a length scale comparable with that of the enamel rods.

Cast within the framework of the Finite Element Method (FEM), the model consists of an implementation of the reaction-diffusion differential equations within a Representative Volume Element (RVE) that reflects the periodic structure of rod and sheath domains within human tooth enamel. The first attempt at modelling the physics of this problem was proposed in 1966 by Zimmerman [13], who included up to seven different reactants within the partial differential equations in the 1D domain. Similar attempts were made in the years that followed, however the role of diffusivity anisotropy and realistic geometrical features of the modelling framework were not accounted for [14], [15], e.g. prisms or teeth geometrical shapes. Additionally, to the best of the authors’ knowledge all similar type mathematical approaches found in the literature lack experimental validation [16], [17]; except for a relatively recent paper that studies the dissolution at the micron scale without accounting for the enamel local anisotropy properties [18]. The key novelty of the present study lies in the fact that demineralisation during conditions of artificial caries is modelled at the level of enamel rods with full consideration of the local HAp crystal orientation. To produce a reliable model of this process, numerical calculation results were validated against experimental observations of the phenomena. Advanced high-resolution experimental techniques employed for this task were synchrotron X-ray micro-Computed Tomography (µCT) and Scanning Electron Microscopy (SEM), as well as Transmission-SEM (STEM). In particular, the observation of enamel rods erosion enabled quantitative statistical information to be obtained that was essential for the comparison experiment vs. model results for the purpose of validation.

Additionally, the effect of the etching attack acting with respect to the rod longitudinal axis on the etched surface morphology needs to be investigated. To shed some light onto this aspect of the process and to demonstrate the power of the newly developed analysis tool, a simplified simulation was carried out with etching surface being normal to the rods’ longitudinal direction. On a qualitative basis, the impact on the acidity of the etchant was also evaluated, using the model through parametrical analyses at several levels of pH, and discussed below.

## Material and experimental methods

### Sample preparation

In this study, an intact human third molar was used that was extracted for therapeutic reasons other than caries (National Research Ethics Committee; NHS-REC reference 09.H0405.33/Consortium Reference BCHCDent332.1531.TB). The root tips of the analysed tooth were removed using a rotating diamond saw at very low speed to avoid microstructural modifications. After that, the molar was fixed in 10% formalin buffered solution (Sigma Aldrich, UK) for four days. Subsequently, the sample was rinsed with water and sectioned to obtain 500 μm-thick slices.

The tooth slice was embedded in a bespoke envelope made of Kapton sheet and nail varnish, in order to allow only for the natural tooth free surface to be exposed to the etching agent. The varnished tooth slice was incubated at 37 °C in lactic acid (0.5% v/v, 0.5 mL) to simulate the carious process [19] for three weeks at pH 4.4, whilst deionised water with pH 7.0 was used as control. Solutions were changed every two days to ensure the environment was maintained.

## X-ray μCT experiment

The sample was examined *ex situ* exploiting the X-ray beams generated at the UK synchrotron facility, Diamond Light Source (DLS, Oxford Harwell Campus, Didcot, UK). The experiment was conducted on the I13-2 beamline dedicated to imaging and tomography studies. Synchrotron X-rays facilities provide unrivalled compromise between x-ray flux and spatial resolution achievable [20].

X-ray micro-computed tomography (X-ray μCT) was performed using a pink X-ray beam with moderate energy spread. The average energy of the X-ray beam employed was 21.8 keV. To accomplish this task, a set of absorption contrast images were taken in transmission mode at different sample rotation angles with respect to the incident X-ray beam, ranging from 0 to 180°, with an angular step of 0.05°. Each image was acquired at room temperature using an exposure time of 0.3 s. Full data acquisition for the entire set of sample rotations took less than 30 min. To acquire the transmitted radiographs, a PCO Edge 5.5 camera (PCO AG, Germany) was positioned at a distance 940 mm downstream from the sample. The imaging detector is equipped with a CMOS sensor with a 10x objective that provided the pixel size of 0.325 × 0.325 μm (so that the reconstructed data had an isotropic voxel size of 0.325 μm), and the field of view of 0.83 × 0.70 mm. The distortion correction of the acquired images was performed prior to the reconstruction using an array of points with known position. Dark and flat field images were also captured prior to the acquisition of a sample to eliminate noise due to pixel sensitivity variation and other background artefacts. Subsequent processing for 3D reconstruction was performed using SAVU processing pipeline (DLS, UK).

The analysis of reconstructed 3D data from X-ray μCT imaging was performed using Avizo v2020.1 software (Thermo Fisher Scientific).

The full dataset contained 2510 × 2510 × 2110 voxels. To reduce the computational cost, detailed analysis of the features of interest was conducted on sub-volume of 1380 × 850 × 861 pixels (448.175 × 275.925 × 279.500 µm). De-noising processing step was applied using a median filter and a non-local mean filter in Avizo software. For image processing, the background was removed via watershed thresholding and manual segmentation. For the purpose of tracking the eroded rods and therefore the lesion advance, segmentation was performed on the filtered data with the segmentation editor. The statistical information was extracted for the evolution of volume of segmented areas as a function of the distance from the enamel surface.

## FIB sectioning and SEM & STEM imaging

Morphological investigation of the etched surface was conducted by means of electron microscopy using FIB-SEM-STEM technique at the Multi-Beam Laboratory for Engineering Microscopy (MBLEM), Department of Engineering Science, University of Oxford (Oxford, UK). Some examples of previous applications of these techniques in biomaterials are given in [21].

Due to the low sample conductivity, the surface was coated using a magnetron sputtering technique to produce a thin Au-Pd coating layer of 5 nm thickness, thus improving the imaging resolution achievable to a few nm. Due to the local roughness of the sample it was expected that the coating layer thickness could experience some deviation from the nominal value without affecting the imaging results. In addition, STEM imaging was carried out at a position close to the fully demineralised region of the enamel. A STEM lamella sample was extracted from the tooth by exploiting the capabilities of Tescan LYRA instrument (Tescan s.r.o., Brno, CZ). Focused Ion Beam (FIB) was used together with the Gas Injections System (GIS) and nanomanipulator for sample lift out. The lamella preparation procedure produced lamella of ~100 nm thickness thin enough to resolve individual HAp crystallites.

## Experimental results

### Synchrotron X-ray µCT

**Fig. 2:**
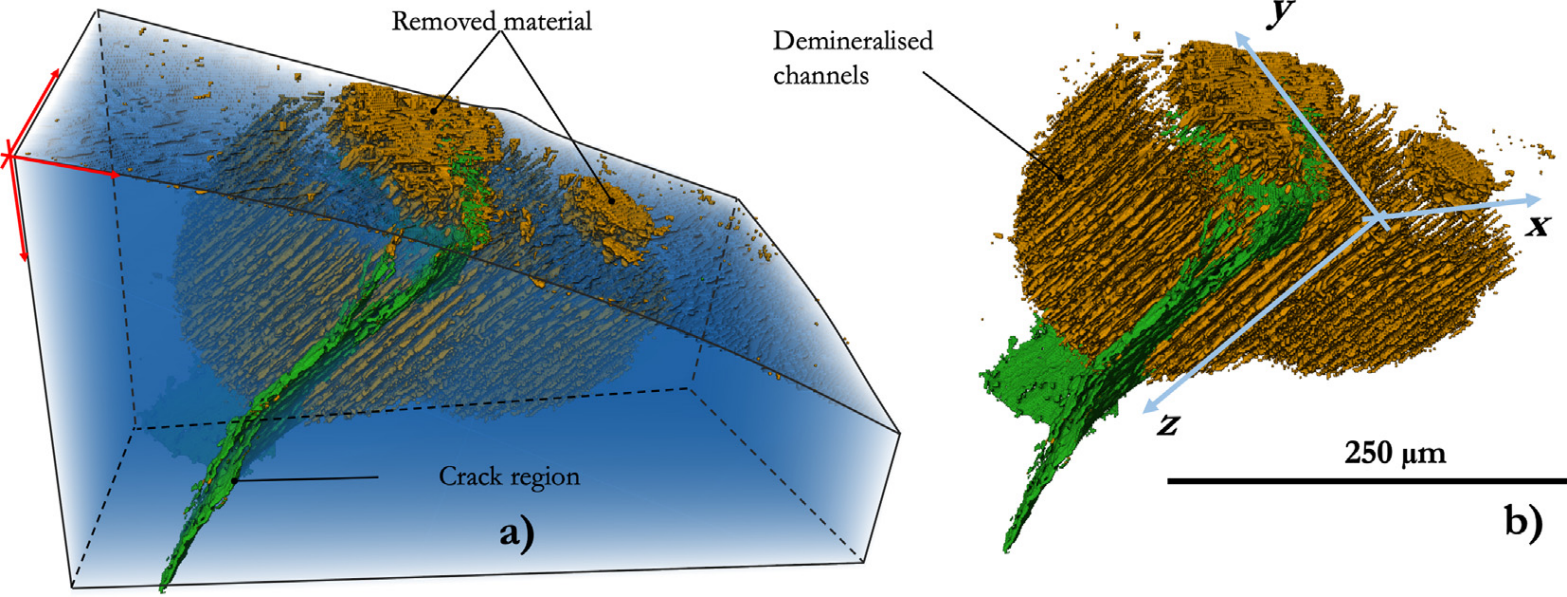
3D reconstructed volume of a demineralised enamel (colour online). (a) The region shown in blue is pristine enamel. Orange channels represent the demineralised enamel rods and the complete material removal (cavity formation). The region shown in green represents an empty volume (after thresholding) that correlates with an enamel fracture. (b) Visualisation excluding the surrounding pristine enamel.

The result of the segmentation of 3D tomographic reconstruction of the artificial caries lesion is shown in Fig. 2. Three different regions can be distinguished, namely the unmodified enamel (blue colour), the eroded enamel rods (orange colour) and the presence of cracking and voiding in the middle of the lesion (green colour). The green highlighted region also includes the domain within which the enamel was demineralised to such low density state that its overall strength was insufficient to hold the material together, and therefore was completely detached and removed from the bulk sample. The presence of these distinctly discontinuous features could be easily identified during segmentation and neglected during subsequent statistical analysis of the erosion of enamel rod structure.

By performing the segmentation of the eroded enamel rods, in a selected sub-volume of the whole probed sample, it was possible to extract the statistical data for the demineralised area fraction as a function of the depth from the sample free surface subjected to acid treatment. The numerical values obtained from this post-processing analysis are reported in the section of this paper below that is dedicated to the comparison with numerical predictions.

### SEM & STEM imaging

In order to obtain further insights and confirm the nature and orientation of the HAp crystallites, SEM and STEM investigations were carried out within the sample.

**Fig. 3:**
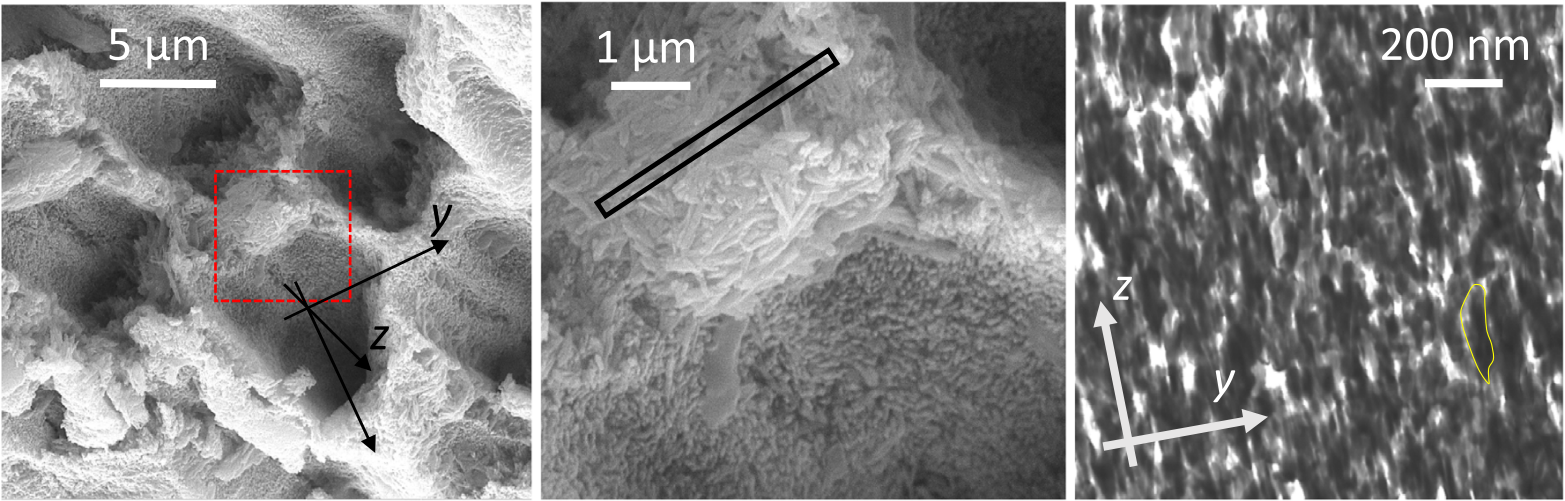
SEM and STEM imaging of etched enamel. (a) SEM etched enamel rods overview. (b) SEM close-up to the rod sheath and interrod enamel for the area indicated by red dashed box in (a). (c) STEM image of the HAp crystallites, in yellow a crystal highlighted. The STEM lamella was extracted from the region highlighted by the rectangular box in (b).

Fig. 3 is a series of electron microscopy images showing the demineralised rods obtained at different magnifications using different techniques. It is possible to observe from Fig. 3(a) that the hollow structures were associated with the demineralised enamel rod head, while the surrounding continuous solid material belonged mainly to the interrod and sheath regions, as well as the sheath regions that appeared to be less affected by the acidic etchant. These observations lead to the conclusion that the etching mode manifested in the present study could be identified as predominantly Type 1. A close-up of Fig. 3(a) for the region outlined by the red box is shown in Fig. 3(b). At this magnification the HAp crystallites were clearly visible, especially for those in the enamel interrod regions. It is worth noting that the crystallites showed preferential morphological orientation, i.e. their larger extent lay within the plane approximately normal to the rod elongation. This was an important observation since, as discussed earlier, the orientation of the HAp crystallites had a strong influence on diffusivity and the mode of demineralisation advance. The result of this observation in combination with the insights into the orientation distribution provided by the body of literature was implemented in the numerical model presented in the following sections of this paper.

A STEM image of the partially demineralised enamel is shown in Fig. 3(c). The lamella was extracted from the interrod region of the rod after demineralisation, hence the STEM image appears to be highly porous. HAp crystallites appeared to be shorter in length compared with values cited in the literature and also compared with SEM assessment from images similar to Fig. 3(b). Although it was tempting to attribute this to the effect of dissolution during etching, in practice this was likely to be the consequence of the lamella thinning procedure using FIB. Remarkably, the transverse size of the crystals remained consistent with the size seen in the SEM images. This indicated that there exists significant anisotropy in terms of the chemical bonding and mechanical strength as a function of orientation within HAp crystals.

## FEM modelling of enamel dissolution

FEM methods are nowadays employed in a number of numerical analyses and simulations to solve differential equations in complex shaped domains. Due to its great versatility, FEM fields of application span from structural to biological, from thermal to optical and so on [22], [23], [24], [25], [26], [27]. Often, a multiphysics approach is sought by coupling and solving problems governed by different physics as well. Of particular interest is the use of FEM modelling for structural purposes, for example the effect of restoration [27] and masticatory forces [28].

### Fundamental equations and RVE description

In this work, the dissolution of HAp crystals was considered to be a first-order kinetic process driven by the action of a single component in solution, i.e. the concentration of hydrogen ions, H, was chosen as the key variable. Within the domain, the change of hydrogen ion concentration, H, over time was expressed by Fick’s second law modified to account for the production of the hydrogen ions by the solution reaction. Therefore:

(1) $$\frac{\partial H}{\partial t}=\nabla \left[\bar{\bar{D_{G}}}\left(x,y,z\right)\nabla H\right]+R_{\mathrm{diss}}.$$

here the derivative of H with the respect to time represents the accumulation rate defined in the geometrical domain with respect to a global coordinate system with position x,y,z and time t. On the right-hand side of the differential equation, the first term corresponds to the flux gradient in which $\bar{\bar{D_{G}}}\left(x,y,z\right)$ is the effective diffusivity hydrogen ions in the enamel domain. The double overbar indicated that the anisotropic diffusivity is thought of as a 3 × 3 matrix defined in the global coordinate system.

The nature of the hierarchically structured material studied here was such that at the small scale of consideration involved the mineralised dental tissue could not be considered homogeneous, and its diffusivity response was not isotropic. For the correct calculations to be performed, it was necessary to implement the rotation of the diffusivity matrix from the orthotropic form at the micron-scale aligned with the local rod orientation to the global coordinate system. Further correction to the diffusivity tensor was required to account for the evolving porosity that affected material permeability. The former issue will be addressed in the section devoted to this issue below, while the considerations required to incorporate the porosity effect are presented here. In mass transport problems, material porosity is known to affect the overall diffusivity of a component within a structured host material [29]. If the intrinsic diffusivity coefficient of an elemental homogeneous material domain is $D^{*}$, the effective diffusivity of a porous solid in a local coordinate frame can be expressed as:

(2) $$\bar{\bar{D_{L}}}=\frac{D^{*}\varepsilon_{t}\delta }{\tau },$$

where $\varepsilon_{t}$ is a dimensionless parameter indicating porosity as the percentage of cross-sectional area available for transport, δ is constrictivity, and τ is tortuosity. However, in the specific problem addressed in the present study the precise diffusivity $D^{*}$ of Hydrogen ions in HAp crystals is unknown. Furthermore, the parameters listed are presently not known due the limitations of the existing experimental techniques available to investigate proton transport at small length-scales down to the nanometre level. To overcome these issues, the matrix $\bar{\bar{D_{L}}}$ was calibrated in the model against the experimental evidence of the time-dependent experimental test to determine the effective diffusivity as a set of homogenised parameters for the particular material configuration in which the orientation of the underlying constituent HAp crystallites is known. The full description of the local diffusivity tensor $\bar{\bar{D_{L}}}$ and the derivation of the global diffusivity tensor $\bar{\bar{D_{G}}}$ can be found in the following section, with further details given in the Appendix.

It is important to mention that diffusivity is a temperature-dependent parameter whose variation can be expressed by the Arrhenius equation:

(3) $$\bar{\bar{D_{L}}}=\bar{\bar{D_{O}}}e^{\left(-\frac{E_{A}}{\mathrm{RT}}\right)}.$$

Here $\bar{\bar{D_{O}}}$ the maximum value of the diffusion coefficient (at infinite temperature), $E_{A}$ is the activation energy for the diffusion process, R is the universal gas constant and T is the absolute temperature. In any case, the temperature dependence is not an issue that was faced in this study.

The second term on the right-hand side of Eq. (1) is the dissolution component, $R_{\mathrm{diss}}$. It is defined as:

(4) $$R_{\mathrm{diss}}=k\left[H_{c}-H\right],$$

where $H_{c}$ is the apparent solubility of HAp in the bulk solution that is a function of the solution ion activity, and k(x,y,z,t) is a first-order reaction surface rate constant. This linear relationship was found to be a good approximation for this type of process [17].

As concerns the discrimination of the demineralised regions over the time, the approach adopted here makes the use of the level-set phase-change model. This approach is preferred over the method that considers element deletion because it avoids deactivation of elements over the time, therefore the mesh remains the same during the time-dependent simulation.

At the length scale considered, a HAp crystallite can be considered to be either pristine or completely dissolved. To achieve numerical stability, the phase parameter Φ is introduced equals 0 when all HAp crystals occupying an elemental volume are fully dissolved, and equals 1 when the entire elemental volume is filled with undamaged HAp crystals. A threshold value of parameter Φ can then be used for the identification of the demineralisation front. The differential equation governing the spatial and temporal evolution of Φ is written as:

(5) $$\frac{\partial \Phi }{\partial t}=\gamma \nabla \left[\varepsilon \nabla \Phi -\Phi \left(1-\Phi \right)\frac{\nabla \Phi }{\left|\nabla \Phi \right|}\right].$$

Here the terms on the left-hand side describe the motion of the interface in terms of the partial time derivative. The terms appearing on the right hand side are necessary to ensure the stability of the numerical solution. Parameter ε defines the thickness of the region over which Φ changes its state from 0 to 1 and vice-versa in a smooth transition. For numerical purposes, this parameter is fixed to be equal to the element size of the discretising mesh of the FEM model. Constant γ is used to reinitialise and stabilise the level set function; it is important tune it in order to avoid unwanted oscillations of Φ in the solution.

### RVE description

**Fig. 4:**
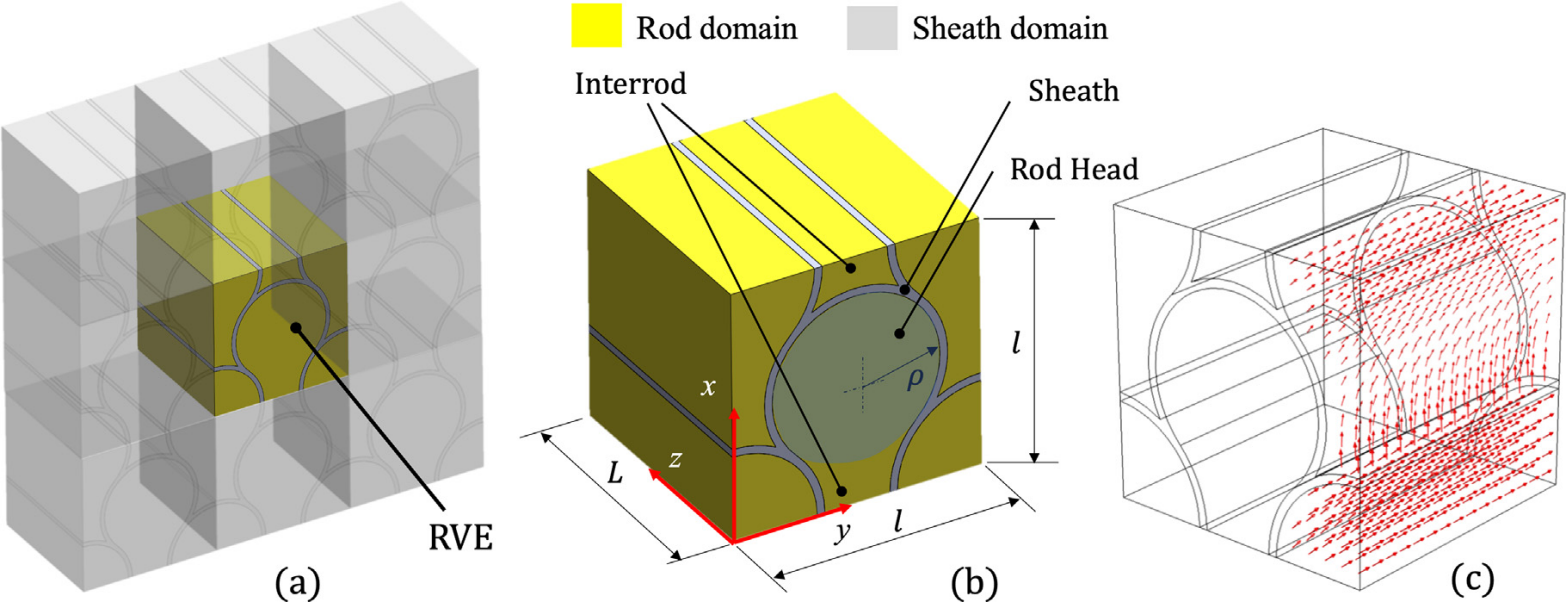
Representative Volume Element characteristics. a) Periodicity representation and extraction of the RVE. b) Geometrical characteristics and relevant domains, along with a cartesian coordinate system. b) Hydroxyapatite crystals orientation indicated by arrows.

The above equations were solved within the finite element framework over a three-dimensional domain representing the periodical structure found in the tooth enamel. The software package used in this work was COMSOL. As illustrated in Fig. 4, the RVE is taken as a square cross-section *l × l*, extruded normally to its own plane by the length *L*. In the model described here *l* was set equal to 7.1 µm, and *L* was chosen to be equal to 250 µm, so that with the dimensions given in micrometres, the RVE domain can be written mathematically as:

(6) $$V=\left\{0<x<7.1,0<y<7.1,0<z<250\right\}$$

The choice of the large ratio *L/l* ~ 35 was dictated by the experimentally observed high diffusivity of H ions in the *z*, direction (along the rod) compared with the corresponding values in the *x* or *y* direction, µCT observation. The well-known keyhole-like geometry of the enamel rod was reproduced in the RVE model as shown in Fig. 4, with the characteristic length of the rod head identified with a radius ρ = 2.4 µm. The thickness of rod sheath was assumed to 0.22 µm. The way of prescribing the HAp crystal orientations within the RVE is the topic of the next section.

In the context of the FEM analysis, the discretization of the RVE was performed by firstly meshing the 2D plane *xy* with a mapped method and then sweeping the elements along the longitudinal direction *z*. This permitted a very low degree of element distortion while maintaining a great mesh uniformity, ideal to minimise numerical errors.

### Boundary conditions

Eq. (1) was solved assuming periodic boundary conditions for the concentration along axes *x* and *y*, namely, periodic boundary conditions$H_{y=0}=H_{y=7.1}$, were applied over the surfaces having *y* as the normal axis, and $H_{x=0}=H_{x=7.1}$ for the surfaces having *x* as normal axis.

In widespread practical use, pH is used as the parameter to describe hydrogen ion concentration according to the following relationship:

(7) $$H=10^{-pH}$$

An initial concentration of $H=10^{-14}$ *Mol/litre* was set within the domain *V,* corresponding to pH = 14*.* Same value of concentration was kept fixed throughout the time-dependent simulation at the flat surface having *z-axis* as the normal axis ($H_{z=250}$). Lastly, the excess of hydrogen ions created at the outer free surface of the enamel due to the presence of the acid is also modelled by applying a Dirichlet boundary condition over the plane *xy* at z = 0, i.e. $H_{z=0}=10^{-{\mathrm{pH}}_{\mathrm{acid}}}$*.*

A single set of algebraic equations was generated corresponding to Eqs. (1), (5), together with the boundary conditions applied to volume *V*. A fully coupled method was generated to implement the equations in a single iteration scheme solved until the convergence condition is reached.

In order to track the demineralisation process over the time, the mineral content was evaluated in cross sectional planes *xy* perpendicular to the *z* direction for each time step. To do so, the phase-field parameter Φ was employed as follows:

(8) $$M\left(z,t\right)=\underset{A}{\int^{\;}}\Phi \left(x,y,z,t\right)dxdy.$$

Here $M\left(z,t\right)$ is the time evolution of the mean mineral content in cross-sections perpendicular to the *z*-axis, evaluated by computing the double integral of the phase-field parameter Φ(x,y,z,t) across the cross sectional area *A*. This parameter correlates well with the apparent density of the material that can be directly compared with the experimental findings once properly scaled and normalised.

### The implementation of anisotropic diffusivity related to crystallite orientation

As observed in previous studies [10], the diffusivity of solutes within HAp crystallites shows a markedly anisotropic behaviour. The diffusivity coefficients within HAp platelets within bones were found to be proportional to crystal length in the direction of diffusion [30], as the diffusion of H+ ions through HAp crystals occurs preferentially in the long direction of the crystal (the c-axis) [10].

**Fig. 5:**
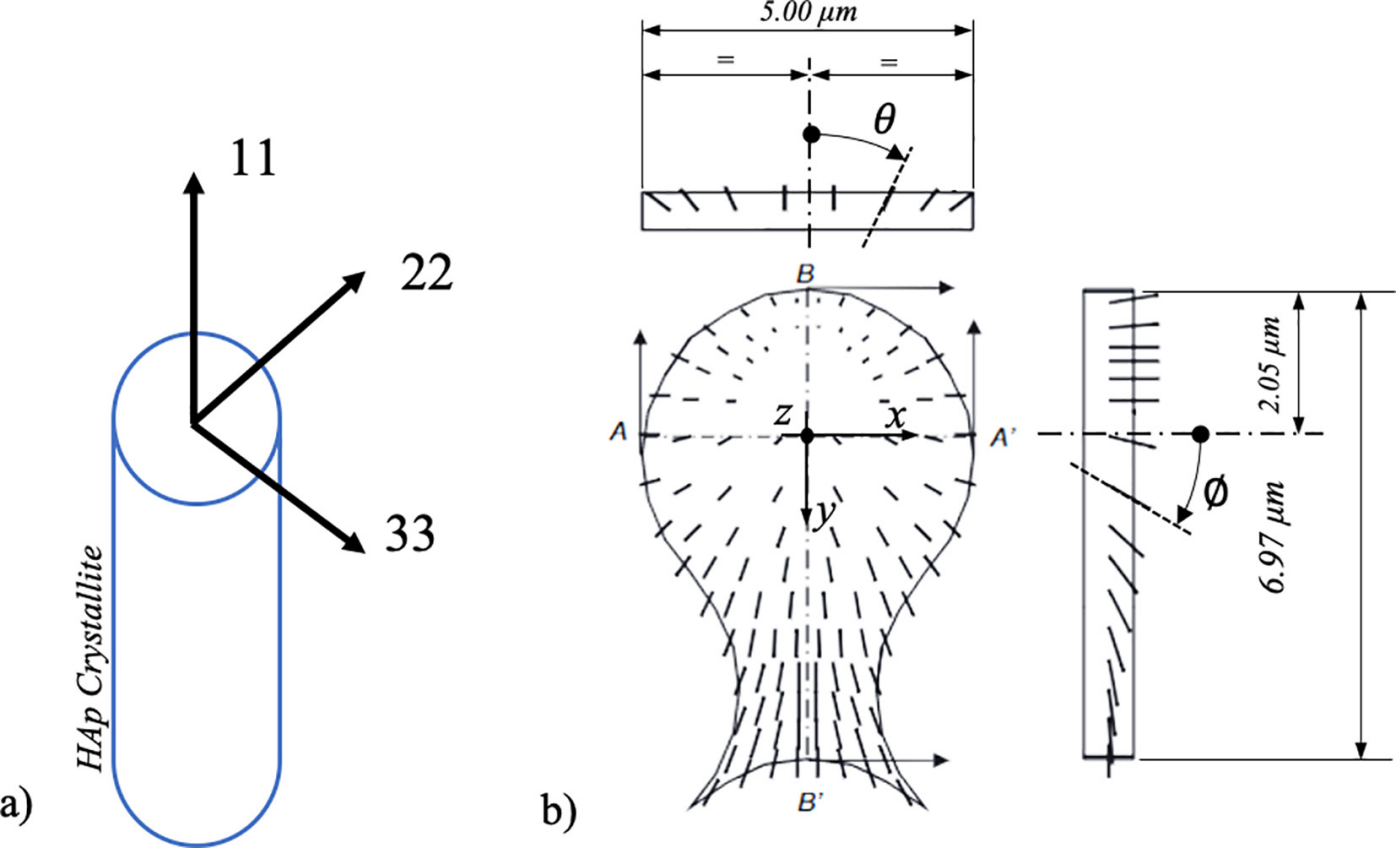
HAp schematisation. Crystal axes and rotation angles. Reproduction from [31]

In this work the *c*-axis of the HAp crystal is identified with the local coordinate axis 1, while the *a*-axis and *b*-axis are identified with the coordinate axes 2 and 3 respectively, as illustrated in Fig. 5.

In accordance with Fig. 5, the second-order local diffusion matrix $\bar{\bar{D_{L}}}\left(x,y,z\right)$ is defined as:

(9) $$\bar{\bar{D_{L}}}\left(x,y,z\right)=\left[\begin{array}{ccc} d_{11} & 0 & 0\\ 0 & d_{22} & 0\\ 0 & 0 & d_{33} \end{array}\right]$$

**Fig. 6:**
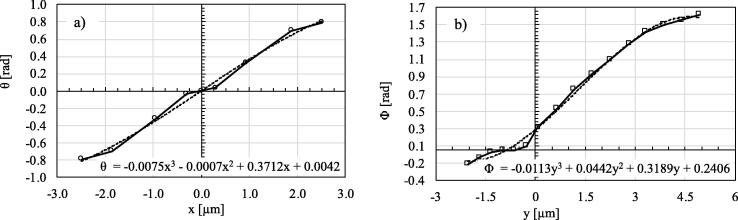
Two angles defining the positive orientation at the element centroids, around directions x and y. The dashed lines indicate the fitted polynomial function which has been implemented in the FEM modelling.

**Fig. 7:**
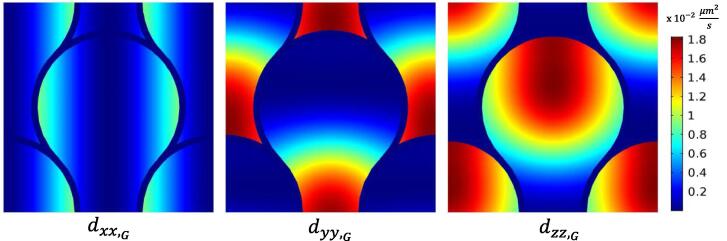
Contour plots of the diagonal elements of the rotated diffusivity matrix $\overset{Ì¿}{D_{G}}$.

In the local coordinate system aligned with the principal directions of diffusivity, $\bar{\bar{D_{L}}}\left(x,y,z\right)$ appears as a diagonal matrix. Given the symmetry of the crystal structure, diffusivity values along directions 2 and 3 are assumed to be the equal [30], $d_{22}$=$d_{33}$. The operation that allowed for the determination of the $\bar{\bar{D_{G}}}$, is reported in the Appendix of this paper. This calculation required as inputs the angles Θ and Φ according to Fig. 5. This information was extracted from the literature [31]. In order to smoothly prescribe such a distribution within the mathematical model, third order polynomial function fittings were employed, as shown in Fig. 6. Contour plots of the diagonal elements of the diffusivity matrix $\bar{\bar{D_{G}}}$, prescribed within the *xy* planes along the *z*-direction, are shown in Fig. 7. Note that $\bar{\bar{D_{G}}}$ presents also non-zero coefficients which are not shown here, i.e. $d_{\mathrm{xy}}$*,* $d_{\mathrm{xz}}$ and $d_{\mathrm{yz}}$ (Please refer to the Appendix for the determination of $\bar{\bar{D_{G}}}$). The six off-diagonal terms describe the correlation of random motions of H ions between each pair of principal directions (i.e. xy, xz, yz).

The calibration procedure of the model made use of the experimental data captured after 3 weeks of exposure of a healthy molar to lactic acid at pH 4.4. Along with the initial fixed parameters it was assumed that the pristine enamel contained a negligible amount of hydrogen ions, therefore the initial *pH* within the analysed domain was set to 14. It is well documented that the demineralisation process initiates once the *pH* of the attacking acid falls below the threshold value of pH 5.5 [32]. This value was set as the boundary for the phase change condition, so that enamel demineralisation begins once the concentration of H ions exceeds the corresponding value. It is worth mentioning though that this critical value may vary depending upon the concentrations of calcium and phosphate in the saliva and plaque fluid [33].

The other parameters that needed to be sought and set to validate the model were the diffusivity of H ions within HAp crystallites in the local coordinate frame ($d_{22},d_{33}$) and the reaction surface rate constant k. These coefficients were not available in the current literature for such particular problem. As concerns the enamel rod domains, error minimisation procedure yielded the values of $d_{33}=1.82\times 10^{-2}\;{\mu m}^{2}s^{-1}$ and $d_{22}=d_{11}=3.25\times 10^{-5}\;{\mu m}^{2}s^{-1}$ (note the significant difference in the magnitudes), while the reaction surface rate was found to be $k=2.00\times 10^{-7}\;s^{-1}$. It is worth to report that for this simulation the reaction term did not affect significantly the outcome of the simulation, as it can be seen from its low magnitude. It will become more relevant when the interaction of more than one chemical element will be simulated, in a future work.

As previously discussed, the sheath region is hypo-mineralised and does not show a clear preferential orientation of HAp crystals. However, also the density of HAp crystals - even if small in ratio - within this region may have different density compared to the rod region, which in turn affects the effective diffusivity in accordance with Eq. (2). In order to model the entire demineralisation process, the coefficients prescribed in this region were set differently from those for the enamel rod. The following diffusivity matrix was prescribed directly with respect to the global coordinate system, which resulted in a good match with the experimental outcomes: $d_{\mathrm{xx},sheath}=d_{\mathrm{yy},sheath}=d_{\mathrm{zz},sheath}=6.50\times 10^{-7}\;{\mu m}^{2}s^{-1}$. As concerns the $d_{33}$ coefficient of the diffusivity matrix, this is the only one that can be critically compared to the values found in the literature, given the great difficulty in probing with accuracy these parameters. It turns out that the value of this coefficient appeared to be reasonable compared with the range of diffusivity values found in the literature [34]., i.e lying close to the range of $10^{-1}\;-\;10^{1}\;\mu m^{2}s^{-1}$. Unfortunately, to the best of the author’s knowledge, no experimental results are provided by the current literature regarding the diffusivity coefficients of the *22*, *33* directions and relative to the sheath layer.

## Results and discussion

### Experiment vs. Model

**Fig. 8:**
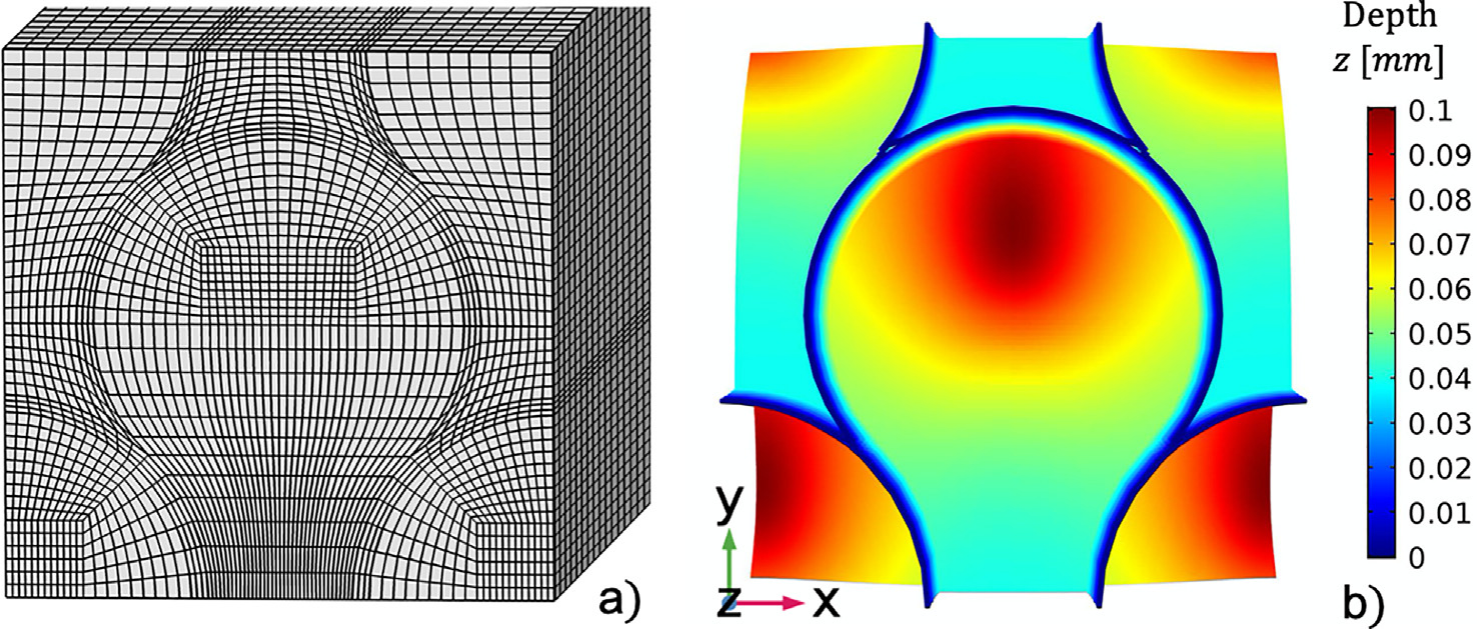
Discretised RVE (a) and an example of the enamel erosion front extracted after 21 days of acid exposure (b).

Geometrical modelling was performed using the discretizing mesh shown in Fig. 8(a). This fine and regular element configuration was chosen to minimise numerical errors. Moreover, a mesh refinement convergence test was performed. This process ensured that the gradients of the resulting variables and the geometrical local curvatures were correctly modelled, while achieving a relatively low computational time. The mesh used for the following simulations consisted of 113,832 elements and linear interpolation functions were used. For the sake of illustration, Fig. 8(b) reports the contour plot of the lesion front corresponding with enamel exposure of 21 days. This helps delineate the regions that were more susceptible to demineralisation, such as the rod heads, whilst the interrod enamel and especially the sheath appeared to be much less affected by the presence of the demineralising acid. These findings were in agreement with SEM observations reported in Fig. 3(a).

**Fig. 9:**
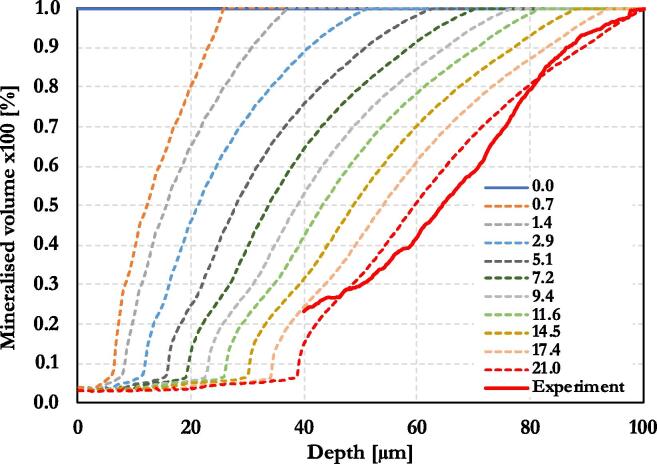
Lesion front change as a function of the time, in days. Modelling vs. experimental data (red solid line).

Alongside the 2D representation seen so far for a determined time, a precise quantification of the demineralisation front advancing was performed as the exposure increased. Fig. 9 summarises the time evolution of the demineralisation simulated up to three weeks of exposure, in a similar manner to what done experimentally; shown as dotted lines. Overlapping with a continuous thick red line is the experimental mineral content found experimentally at the end of the etching process. As can be seen, there was good agreement between the modelling and the experimental trend, for the depth range 40–100 μm.

While the modelling displays a continuous set of profiles ranging from the free-surface interface up to 100 μm, no information regarding the shallow interval from 0 to 40 μm was extracted from the experimental reconstruction. This is certainly related to the loss of mineral adhesion caused by the very porous enamel material that occurred at a mineral content less than about 25%. This material removal effect was not modelled in the simulation because it would not have affected the final results that was of interest to this study. However, it is noticeable that the model captured the sudden loss in mineral content occurring at around 40 μm, which is caused by the complete demineralisation of the interrod and beyond that only the acid resistant sheath remained. It is indisputable that experimental results obtained as a function of the time – other than just the snapshot of what occurred after the three weeks exposure - would have made the validation of the model more robust. Unfortunately, the achievement of temporal evolution of the caries appears to be extremely expensive using synchrotron-base technique, as shown here.

### pH variation analysis

**Fig. 10:**
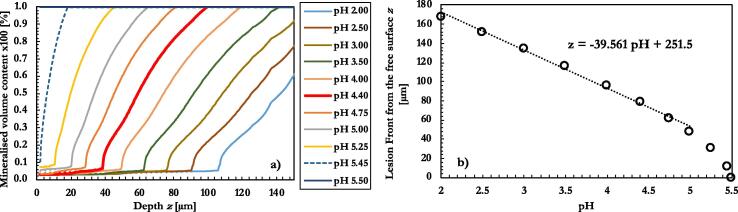
Numerical simulation of the lesion advance. (a) Comparison of exposure to solutions at several levels of pH, after three weeks of continuous exposure. (b) Lesion front as a function of the pH, exposure time three weeks.

Once validated, the model can be very useful for predicting different scenarios, such as pH variations or exposure time. In this section, the effect of different concentration of hydrogen ions in the etching solution was studied using the model. By keeping fixed the exposure time (3 weeks), the etchant acidity was varied from pH 2.0 to 5.5. The plot in Fig. 10(a) shows the demineralised lesion front. As expected, the higher the pH, the shallower the demineralized front.

Aimed at finding relationships between the pH and the front of the lesion, the depth at which the lesion had reached 80% of the mineral content was recorded for each pH and plotted, Fig. 10(b). A linear relationship was found for levels of pH ranging from 2.0 to 5. The same linear relationship was found also in a recent experiment, although the gradient was different [19]. As the pH approached the demineralizing threshold (pH 5.5) the curve assumed a different slope. As expected, at pH values close to the demineralising threshold, the lesion front appeared to be extremely superficial.

### Type 2 scenario simulation

A simplified model was employed in this simulation to improve the understanding the origins of different types of etching patterns. The underlying HAp crystal orientation is thought to affect the direction of the demineralisation front and therefore contribute in determining the type of etching pattern. In order to enhance the visualisation of this phenomenon, this section of the paper considers a rotated etching attack angle (90°) with respect to the Type 1 scenario.

**Fig. 11:**
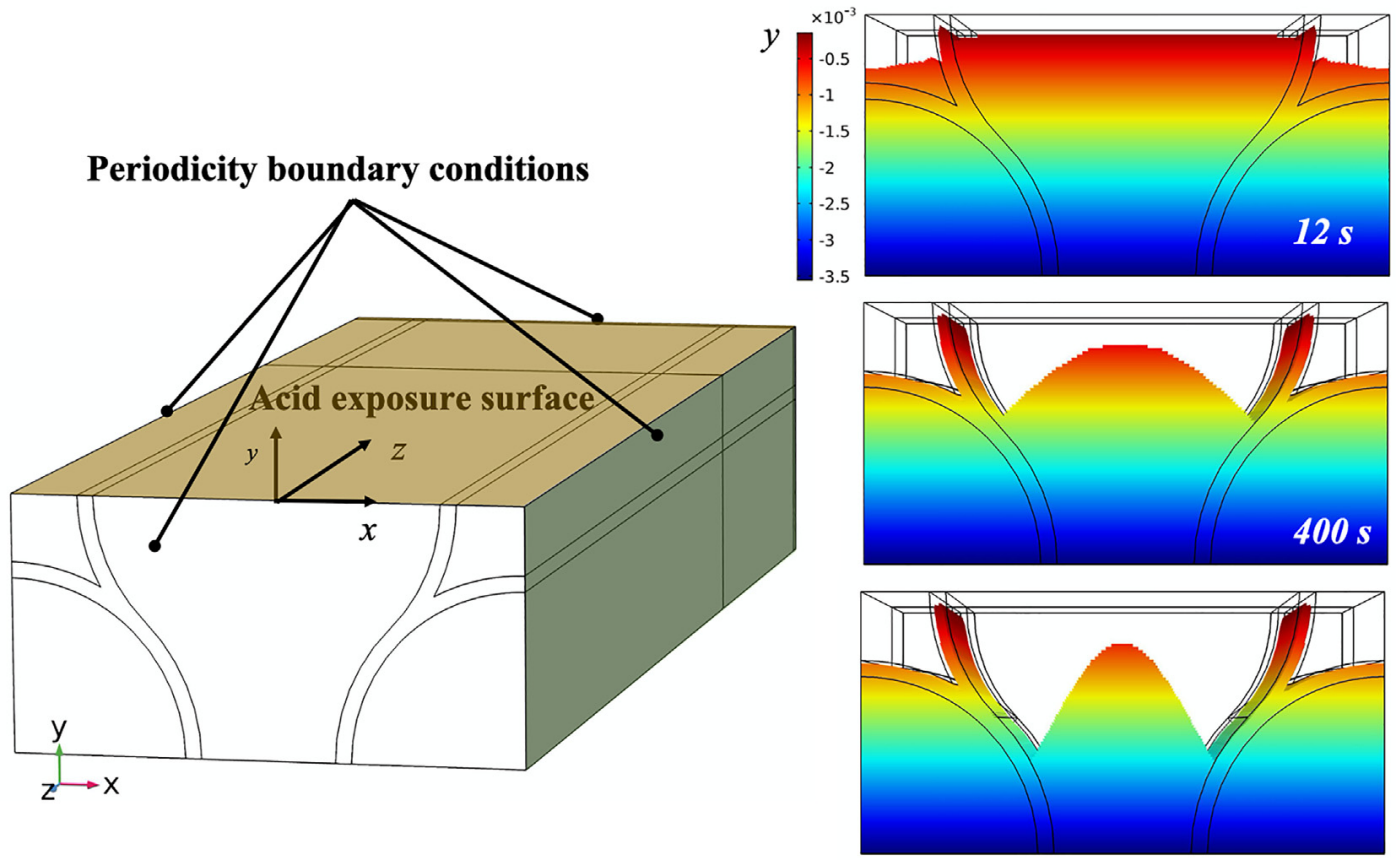
Mixed mode etching type simulation. (a) Geometrical model and exposed surface in blue. (b) Demineralised front at several time frames. The colours represent the y-coordinates for each point of the domain (in millimetre).

To perform this task, the same model as portrayed before was utilised with some modifications in both the geometry and boundary conditions. Firstly, the surface that was the actual source of hydrogen ions was no longer perpendicular to the longitudinal direction of the rod but, normal to that as depicted in Fig. 11. Secondly, the boundary conditions expressing periodicity were applied to the surfaces laying on the planes *yz and xy*. Whilst, on the surface opposite to the exposed one, the boundary condition applied was only the absence of a flux of hydrogen ions. Parameters for coefficients of diffusivity and reaction remained the same as the simulation described in the previous section of this paper concerning the simulation of the experiment, while the etchant pH was set to 5.

In Fig. 11, surface plots of the demineralised front are shown as a function of the exposure time. In this figure, the colours show the depth of the front in the *y*-direction, which can help demonstrate the region where more material had undergone phase change, in other words where it had demineralised. It was revealed that the core of the rod head did not experience deep demineralisation, as seen before in the scenario studied experimentally. Instead, the outer part of the core demineralised more, giving rise to a different etching mode, which was clearly associated with Type 2. Additionally, in the early stage of the demineralisation the interrod regions dissolved first. This simulation is not to say that the etching mode was solely and undoubtedly induced by the acid attack orientation, but that this aspect played an important role [35]. The etchant solution nature and the way it interacts with the enamel substance also affected the etching patterns.

## Conclusions

In this work, the demineralisation process induced by an etchant agent at the micron-scale of human enamel was successfully simulated. For the first time, it was possible to understand and model the dissolution process occurring at this scale and the role played by the HAp crystallites. By accounting for the internal enamel HAp organization and for the disordered and hypo-mineralised content in the sheath regions, an exhaustive agreement was found with respect to the experimental evidence. Only thanks to the micron resolution provided by micro CT using a synchrotron X-ray facility, detailed information about the dissolution progression was observed and therefore directly compared with the simulation at the same length scale. Although the validation was only done at one reference time (after 3 weeks) of the etching process, and although the model only considered the effect of hydrogen ions diffusing and therefore demineralizing the HAp, the model presented here accurately captured the mechanisms involved and resembled the actual morphology observed in both the micro-CT and direct SEM studies.

The model gives some insights into the nature of Type 1 and Type 2 etching patterns. If the enamel rods were exposed to demineralizing agent on a surface parallel to the longitudinal extent of enamel prisms, Type 2 etching pattern was observed. Particularly, the head of the rod appeared to be less prone to hydrogen ion diffusion because of the crystal orientations of HAp crystallites, while the interrod demineralised rapidly. Without claiming this aspect to be fully responsible for determining the etching pattern type, this analysis appears to provide some interesting and thought-stimulating insights.

The novel simulation framework presented hereby constitutes a solid starting point towards the final aim which is to simulate typical tooth demineralization, especially when the tooth is infected by natural caries. To accomplish this task, the capabilities offered by the modelling framework presented here may be fully exploited, for instance, to implement numerous substances that diffuse and react within the enamel, other than simply H ions, e.g. F, P and Ca ions. Other future works may be addressed at transferring the findings of this analysis to a “larger” scale model that would make use of the micro scale diffusion-reaction properties, opportunely homogenised. This would enable the simulation of an entire three-dimensional tooth, with nearly infinite degrees of freedom to analyse a near limitless number of scenarios.

Eventually, it is very important to mention that, this numerical method can also be utilised for the reverse action of the tooth erosion, i.e. re-mineralisation, that general occurs at the enamel top layer thanks to the “healing” action of the saliva.

## Compliance with Ethics Requirements

*In this study, an intact human third molar was used that was extracted for therapeutic reasons other than caries (National Research Ethics Committee; NHS-REC reference 09.H0405.33/ Consortium Reference BCHCDent332.1531.TB).*

## Declaration of Competing Interest

*The authors declare that they have no known competing financial interests or personal relationships that could have appeared to influence the work reported in this paper.*

## Appendix A

In order to achieve the correct definition of the diffusion matrix in the global coordinate system, a base rotation must be conducted. Given the rotation angles provided by the pertinent literature [31], the transformation can be performed as follows.

According to the angles defined in Fig. 5, the rotation matrix can be written as:

(10) $$\bar{\bar{R}}=\left[\begin{array}{ccc} cos(\vartheta ) & 0 & sin(\vartheta )\\ sin(\vartheta )sin(\phi ) & cos(\phi ) & -cos(\vartheta )sin(\phi )\\ sin(\vartheta )(-cos(\phi )) & sin(\phi ) & cos(\vartheta )cos(\phi ) \end{array}\right]$$

Therefore, the base change can be performed by:

(11) $$\bar{\bar{D_{G}}}=\bar{\bar{R}}\bar{\bar{D_{L}}}\bar{\bar{R^{T}}}$$

Which leads to:

(12) $$\bar{\bar{D_{G}}}=\left[\begin{array}{ccc} D11{sin}^{2}\left(\vartheta \right)+D22{cos}^{2}\left(\vartheta \right) & \left(D22-D11)sin(\vartheta )cos(\vartheta )sin(\phi \right) & \left(D11-D22)sin(\vartheta )cos(\vartheta )cos(\phi \right)\\ \left(D22-D11)sin(\vartheta )cos(\vartheta )sin(\phi \right) & {sin}^{2}\left(\phi \right)\left(D11{cos}^{2}\left(\vartheta \right)+D22{sin}^{2}\left(\vartheta \right)\right)+D22{cos}^{2}\left(\phi \right) & \left(D22-D11\right){cos}^{2}\left(\vartheta \right)sin\left(\phi \right)cos\left(\phi \right)\\ \left(D11-D22)sin(\vartheta )cos(\vartheta )cos(\phi \right) & \left(D22-D11\right){cos}^{2}\left(\vartheta \right)sin\left(\phi \right)cos\left(\phi \right) & D11{cos}^{2}\left(\vartheta \right){cos}^{2}\left(\phi \right)+D22\left({sin}^{2}\left(\vartheta \right){cos}^{2}\left(\phi \right)+{sin}^{2}\left(\phi \right)\right) \end{array}\right]$$
